# Verifying the placement and length of feeding tubes in canine and feline neonates

**DOI:** 10.1186/s12917-021-02909-7

**Published:** 2021-06-07

**Authors:** Etienne Furthner, Mariusz Paweł Kowalewski, Paul Torgerson, Iris Margaret Reichler

**Affiliations:** 1 Clinic of Reproductive Medicine, Vetsuisse Faculty, University of Zurich, Winterthurerstrasse 260, 8057 Zurich, Switzerland; 2 Institute of Veterinary Anatomy, Vetsuisse Faculty, University of Zurich, Winterthurerstrasse 260, 8057 Zurich, Switzerland; 3 Institute of Veterinary Epidemiology, Vetsuisse Faculty, University of Zurich, Winterthurerstrasse 260, 8057 Zurich, Switzerland

**Keywords:** Intubation, Ultrasonography, Esophagus, Stomach, Neonate, Milk, Colostrum

## Abstract

**Background:**

Tube feeding is a common procedure in neonatology. In humans, tube misplacement reportedly occurs in up to 59% of all cases and may lead to perforation in 1.1% of preterm intubated neonates. While numerous studies on optimal tube placement have been performed in human neonates, current recommendations on tube feeding in canine and feline neonatology are based, at best, on studies performed in adult animals. Herein, we aimed to test ultrasonography as a tool to verify tube placement in puppies and kittens and to compare different anatomical predictive markers used in human, canine and feline neonates.

**Results:**

The predictive tube length when held bent between the last rib and the mouth may induce trauma compared to when held straight. A strong positive linear correlation was observed between birthweight and gastric cardia localization. Ultrasonography findings were similar to coeliotomy findings. Stomach volume was less than 2 mL per 100 g in the less-than-one-day-old studied puppies (*n* = 25) and kittens (*n* = 28).

**Conclusions:**

A weight-based equation was calculated to help predict appropriate tube placement. Ultrasonography can be used to control gastric tube placement, and neonates less than one-day-old have a smaller stomach capacity. Further studies are required to evaluate whether more-than-one-day-old puppies follow the same linear correlation with their weight. Further in vivo studies are warranted to determine the gold standard procedure for tube feeding in neonatal puppies and kittens.

## Background

In neonatal care, orogastric tube insertion is a common procedure. It allows colostrum or serum intake if canine and feline neonates are unable to suckle colostrum by themselves, preventing enteric diseases, immune deficiency and sepsis [1]. Tube feeding is mostly recommended for normothermic neonates that are too weak to suckle or to be bottle-fed [2–4] and for orphans [5]. Temporary feeding support of neonates is also recommended in cases of lactation failure of the dam, delayed onset of lactation, rejection of one or more of the littermates, too many offspring, mastitis, metritis, or eclampsia [6–8]. Enteral feeding improves gastrointestinal maturation and feeding tolerance compared to parenteral options [9] in both naturally suckling and formula-fed puppies [10]. Tube feeding is a quick procedure compared to bottle feeding and allows better control of the amount of milk given to the puppy [11, 12]. This procedure may be performed by breeders [12, 13] to achieve normal weight gain for all puppies or kittens [14, 15], reducing the time spent compared to bottle feeding, as well as the dam’s energy requirements [7, 11]. Although inserting an orogastric tube in neonates is reportedly a simple procedure [11], multiple complications have been described, such as regurgitations or injuries, leading to bronchopneumonia or even death, with ruptured esophagus and gastric hemorrhage [13, 16]. Regurgitation and aspiration pneumonia are commonly mentioned as a risk of tube feeding [1, 4] and are commonly linked to hypothermia [2, 17], excessive volume [4], speed of feeding [17] and the size of the tube [1, 4, 5]. It has also been suggested that insertion of the tube too deep may create a loop inside the stomach, increasing the risks for regurgitation and trauma [1, 13] or kinking into the gastrointestinal tract [5]. In humans, tube misplacement reportedly occurs in up to 59% [18, 19] of all cases, and perforation due to gastric tubing occurs in 1.1% [20] of low birth weight infants. The most common recommendation regarding the length of insertion in dogs and cats is to measure the distance from the nose to the last rib, slightly bending the tube (BENT) [1, 4, 6, 16, 21–24]. Others recommend using ¾ of that length [5, 11, 17, 25]. To date, recommendations are mostly based on procedures performed in adult animals (esophageal versus gastric tubing) [26–28]. Feeding is repeated multiple times a day in neonates, and radiographic control cannot be performed, contrary to what is done for the gold standard approach in adults [29–31] and in pediatric human medicine [32–35]. Therefore, the measurement of the length of tube inserted is of prime importance. Among common predictive safety measurements performed in human neonates, a nose-earlobe-mid-umbilicus (NEMU) measurement has been described [36–38], as well as weight-based formulas [39, 40]. Correct tube placement may also be assessed under ultrasonography [41, 42]. Other methodologies are also used, such as the auscultation of insufflated air [38], carbon dioxide detection [43, 44] and aspiration of gastric content, all of which pose reliability limitations in neonatology [38, 45].

This study aimed to assess the reliability of ultrasonography control for tube placement in canine and feline neonates to compare different recommendations regarding the length of insertion of feeding tubes using deceased puppies and kittens and to offer a weight-based formula that may help predict cardia location. The maximal stomach volume of puppies and kittens up to 1 day old was also examined.

## Methods

### Animal population

Twenty-eight kittens and 25 puppies that died within the first 24 h after parturition were collected, the informed consent of their owners was obtained to be used for teaching and research purposes. Twenty-five of these kittens and 20 puppies were first stored at − 80 °C and then thawed at ambient temperature for 24 h before measurements. Three feline and five canine neonates that died during delivery or within the first 24 h of life were examined within 12 h after death without being frozen. Only neonates less than 24 h of age were selected based on their history or on the presence of uteroverdin, placenta, placental fluid in the stomach, and umbilical observation [46] whenever more accurate data were missing.

### Feeding tubes and marking

**Fig. 1:**
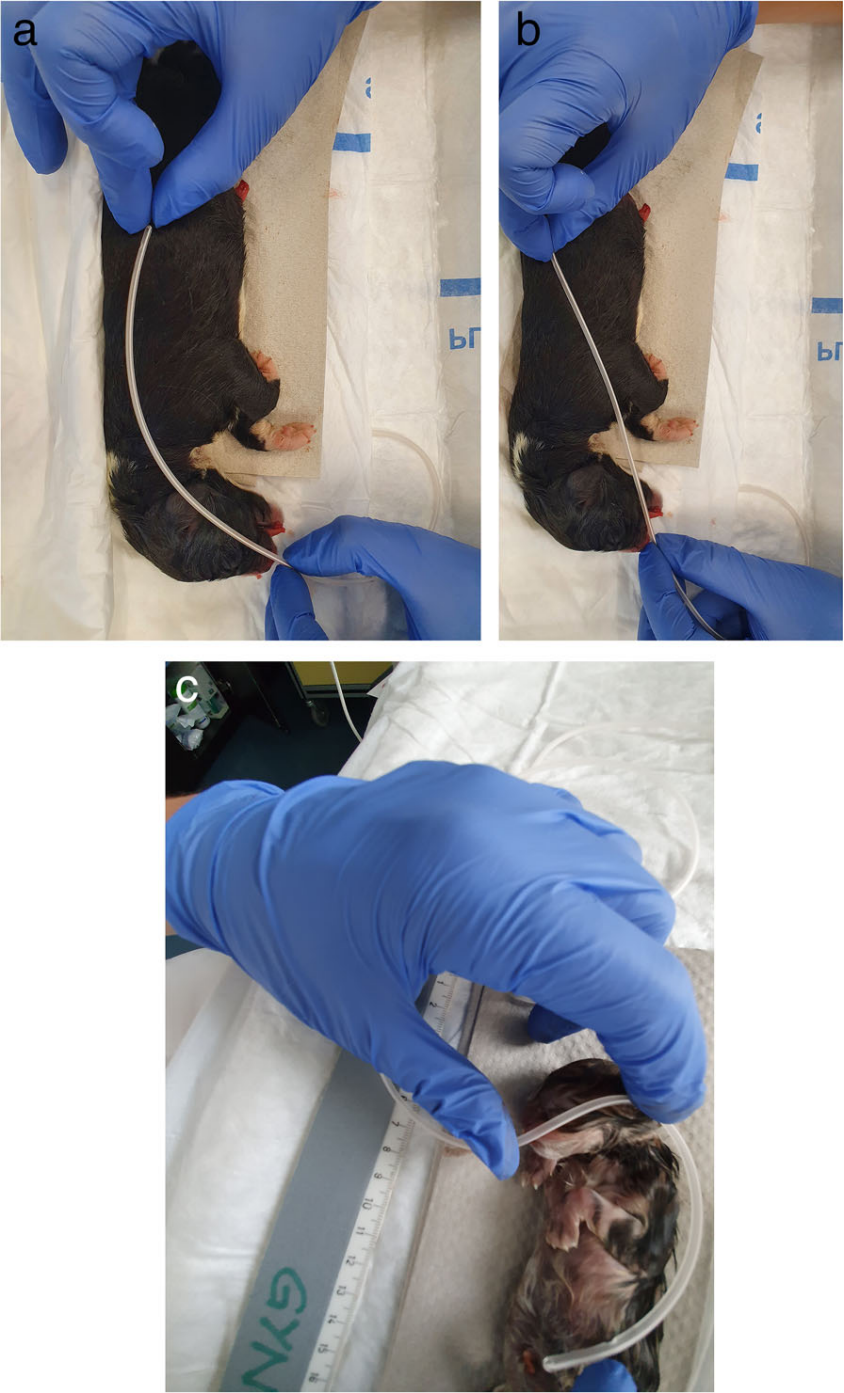
**a** BENT measurement in a neonate. **b** STRAIGHT measurement in a neonate. **c** NEMU measurement in a neonate

Eight Fr diameter, 100-cm-long nasogastric tubes were used (Nährsonde, Medicoplast, Germany). The tube was held alongside the body of the puppy and was bent from behind the last rib to the tip of the nose, and its length was measured (BENT) (Fig. 1a) [6]. Similar measurements were performed between the last rib and the tip of the nose, but with the tube, held straight (STRAIGHT) (Fig. 1b). Finally, we adjusted the NEMU method, which is used to predict the gastric tube insertion length in children [36], to our neonates, using the pinna instead of the earlobe: we recorded the distance from the tip of the puppy’s nose to the pinna and then to the midway between the xiphoid process and the umbilicus (Fig. 1c). BENT ¾ values were calculated based on the three-quarter BENT measurements. These measurements were performed without any markings on the tube to avoid any influence on subsequent measurements. One single operator (corresponding author) measured all parameters of this study.

### Ultrasonography and visual observation

**Fig. 2:**
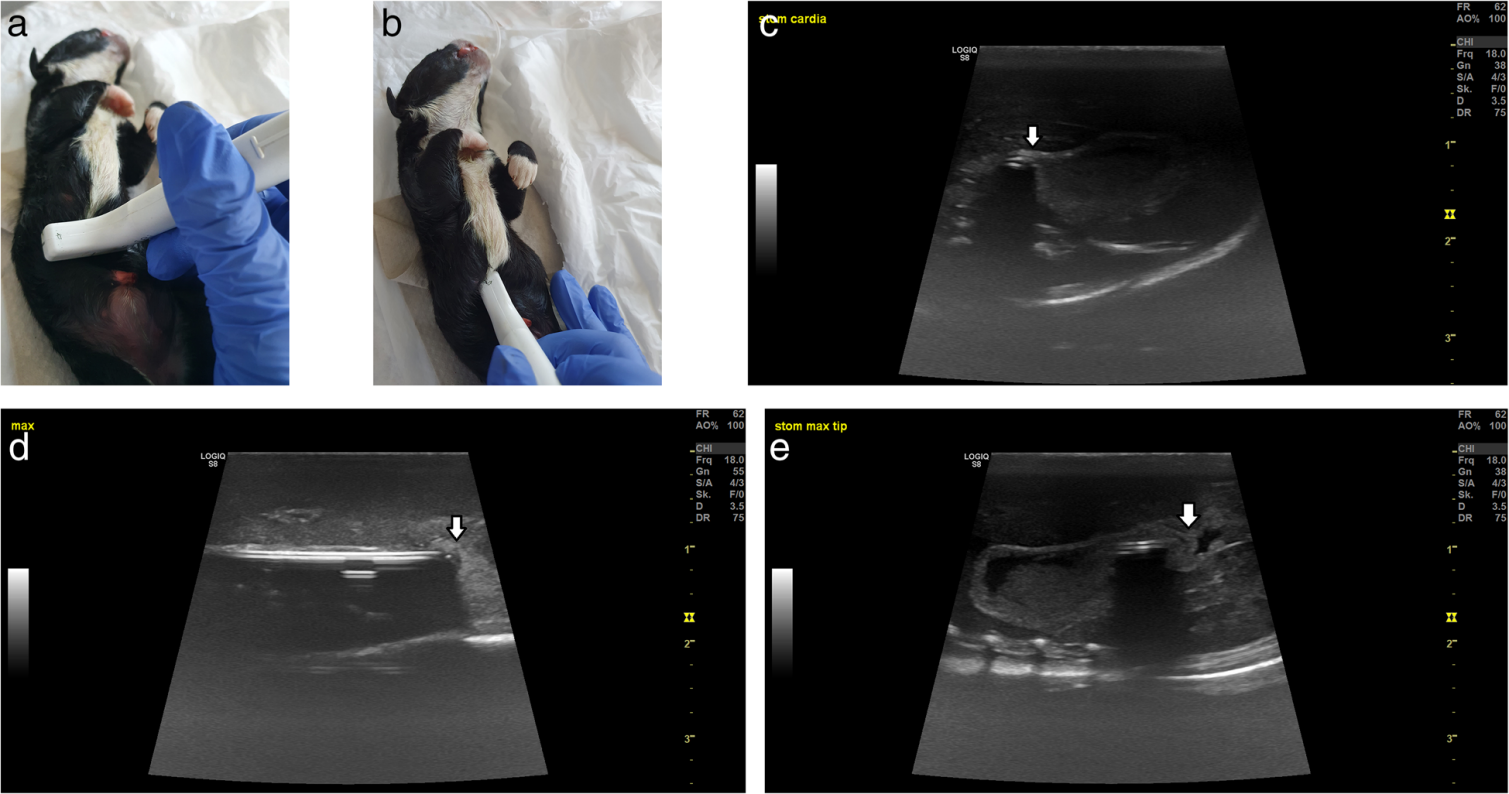
**a** Ultrasonography sagittal scanhead position. **b** Ultrasonography transverse scanhead position. **c** Ultrasound image of the feeding tube entering the gastric cardia (CARDIA US) by ultrasonography. Arrow: tip of the tube entering the cardia. **d** Tip of the tube deforming the gastric wall in an empty stomach (MAX US1) by ultrasonography. Arrow: deformed gastric wall. **e** Tip of the tube deforming the gastric wall in a full stomach (MAX US2) by ultrasonography. Arrow: deformed gastric wall

**Fig. 3:**
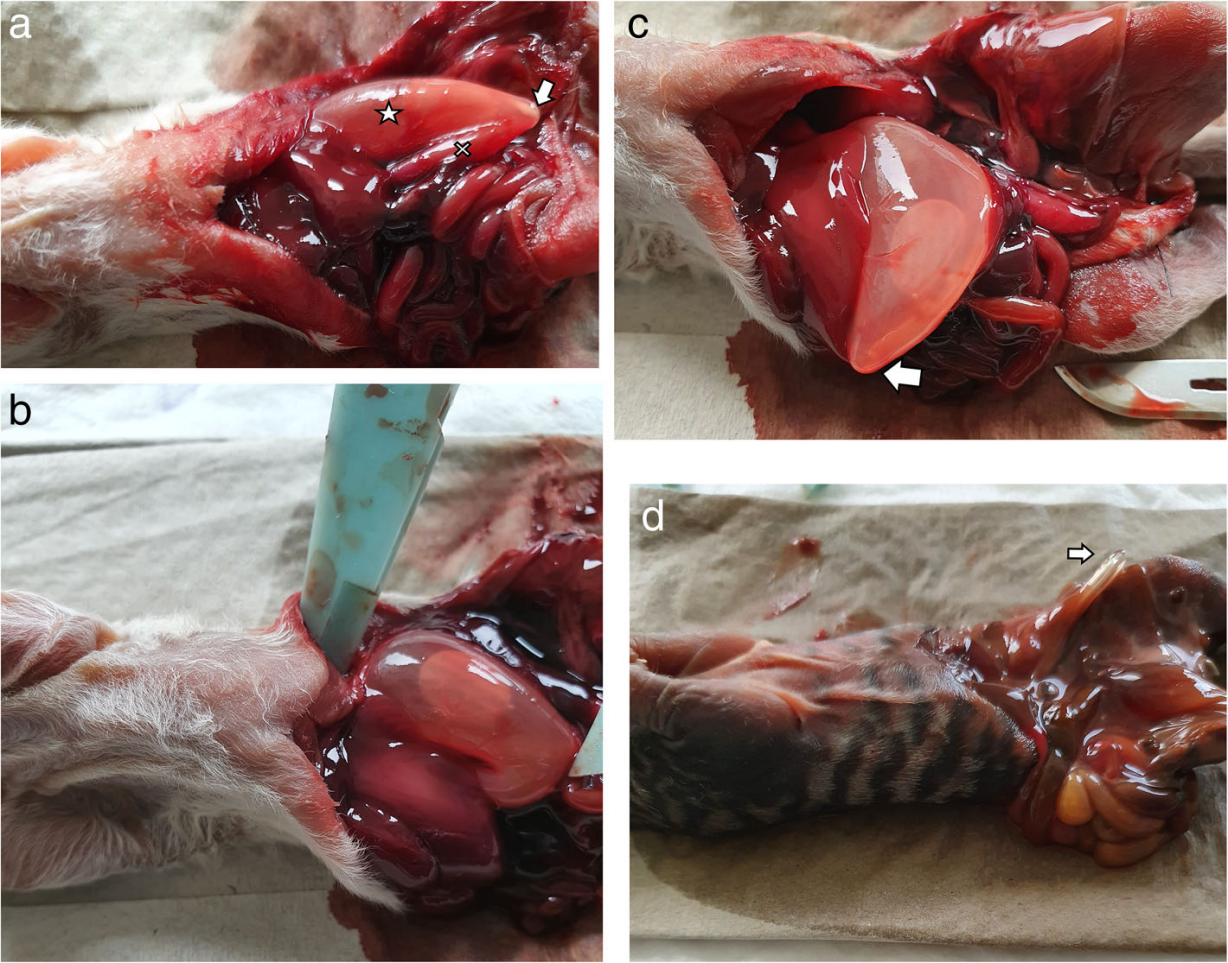
**a** Tip of the tube deforming the gastric cavity (MAX VISUAL) in a neonate after coeliotomy. Arrow: tip of the tube. Star: stomach. Cross: Duodenum. 3b Measurement of stomach volume. **c** Tube looping after forcing the tube further than MAX VISUAL in a neonate. Arrow: tip of the tube. **d** Tube perforating the stomach after forcing further than MAX VISUAL in a neonate. Arrow: tip of the tube

Stomach content and volume (length x width x height) before intubation were evaluated by ultrasonography using GE LOGIQ S8 Vet (Scil, General Electric Healthcare, Switzerland) with a linear 4–15 MHz scanhead. We measured the longest possible stomach length using the transverse scanhead position, together with the width (Fig. 2a). The scanhead was then rotated 90° in the sagittal position (Fig. 2b), where the height of the stomach was measured. Water was used to increase contact and image quality without clipping the hair or using gel. The tube was then inserted adjacent to the palate through the mouth until the tip was visible at the cardia of the stomach by ultrasonography (CARDIA US) (Fig. 2c). The tube was pushed further until the tip touched the stomach and deformed the wall (MAX US1) (Fig. 2d). The stomach was then filled with water at a constant rate of 2 mL/min (120 mL/h) using an automatic infusion pump until the stomach could not expand any further (length x width x height). While the stomach was full, the tip of the tube was pushed further until it touched the stomach and deformed the wall (MAX US2) (Fig. 2e). Then, the abdominal cavity was opened, the length of intubation to the cardia (CARDIA VISUAL) was measured until the tube deformed the stomach wall (MAX VISUAL) (Fig. 3a). The stomach volume was measured visually (length x width x height) (Fig. 3b).

### Statistical analysis

Statistical significance was evaluated using a one-sample two-tailed Student’s t-test of the difference between CARDIA VISUAL and CARDIA US, as well as between STRAIGHT and CARDIA US, using a confidence interval of 95%. *P* < 0.05 was considered statistically significant. Statistical analysis was performed using IBM SPSS Statistics 26 (SPSS Inc., Chicago, IL, USA), and GPower 3.1.9.4 (Düsseldorf, Germany) was used for power analysis. Assumption of normality was tested for skewness and kurtosis, using the Shapiro-Wilk test. Fresh and frozen puppy groups were compared using repeated measures analysis of variance (ANOVA) on the following parameters: the difference between CARDIA VISUAL and CARDIA US and between STRAIGHT and CARDIA US. A regression model was obtained for cardiac length (cm) with respect to weight (g). A *P*-value of < 0.05 was considered significant, and autocorrelation was evaluated using the Durbin–Watson ratio. The results of the linear regression are presented as scatter plots with 95% confidence intervals.

## Results

### Animals

The causes of death of the neonates were a failure to be reanimated after C-section or dystocia, failure to suckle and death or euthanasia within 24 h of life, with three kittens and one puppy that had a cleft palate. The mean weight was 70.2 ± 18.2 g (range 38 to 114 g) for the kittens (*n* = 28) and 216.4 ± 121.9 g (range 67 to 630 g) for the puppies (*n* = 25).

### Tube measurements in kittens

To evaluate the optimal tube length defined as the tip ending in the stomach, regardless of its feeding state without deforming the gastric wall to avoid risks of perforation, BENT ¾, BENT, STRAIGHT and NEMU were compared to CARDIA US, evaluating whether the tip was within the stomach. Then, these values were compared to MAX US1, evaluating whether the tip was within the empty stomach without risk of perforation and to MAX US2, evaluating whether the tip was within the full stomach without risk of perforation.

#### BENT ¾

The tube, when inserted up to BENT ¾, was found in the esophagus in 24/28 kittens at a mean − 0.77 ± 0.77 cm (range − 1.9 to 0.7 cm) cranial to CARDIA US. In the remaining four kittens, it was located in the stomach, never exceeding MAX US 1, the size of the empty stomach.

#### STRAIGHT

The mean difference between STRAIGHT and CARDIA US was 0.23 ± 0.68 cm (range − 1.1 to 1.4 cm) (*P* = 0.11). In 18 kittens, STRAIGHT was longer than CARDIA US values: the tube, when inserted up to STRAIGHT, was found in the stomach in 18/28 kittens and in the esophagus in 10/28 kittens. In one kitten, STRAIGHT exceeded MAX US1, the size of an empty stomach but not MAX US2 (Table 1), the size of the full stomach.

**Table 1:** Number of puppies and kittens where BENT ¾, STRAIGHT, BENT, NEMU or the weight-based formulas were found in the esophagus, the stomach or exceeded MAX US1, MAX US2 and MAX VISUAL

	In the oesophagus	In the stomach (between Cardia US and MAX US1)	Further away than MAX US1	Further away than MAX US2	Further away than MAX VISUAL
**KITTENS (*n* = 28)**					
BENT ¾	24	4	0	0	0
STRAIGHT	10	17	1	0	0
BENT	0	14	14	4	1
NEMU	0	0	28	26	13
Formula Cardia: Ycardia=5.31+2.7*X	16	12	0	0	0
Formula stomach: Ystomach=6.31+2.73X	0	15	13	3	0
**PUPPIES (*n* = 25)**					
BENT ¾	18	7	0	0	0
STRAIGHT	10	13	2	0	0
BENT	0	20	5	4	1
NEMU	0	2	23	23	21
Formula Cardia: Ycardia=7+1.7*X	13	12	0	0	0
Formula stomach: Ystomach=6.31+2.73X	0	14	11	1	0

#### BENT

In 27 of 28 kittens, BENT was longer than CARDIA US and was located in the stomach, while in one kitten, BENT was equal to CARDIA US. The difference between BENT and CARDIA US was a mean of 1.59 ± 1.01 cm (range 0 to 3.4 cm). In 14 kittens, BENT exceeded MAX US1, the size of the empty stomach; in four kittens, BENT exceeded or was equal to MAX US2, which is the size of the full stomach (Table 1).

#### NEMU

NEMU was longer than most of the maximal measurements and would have to be pushed against the stomach wall, at a mean 4.27 ± 1.22 cm (range 1.9 to 6.4 cm), beyond than the cardia.

**Fig. 4:**
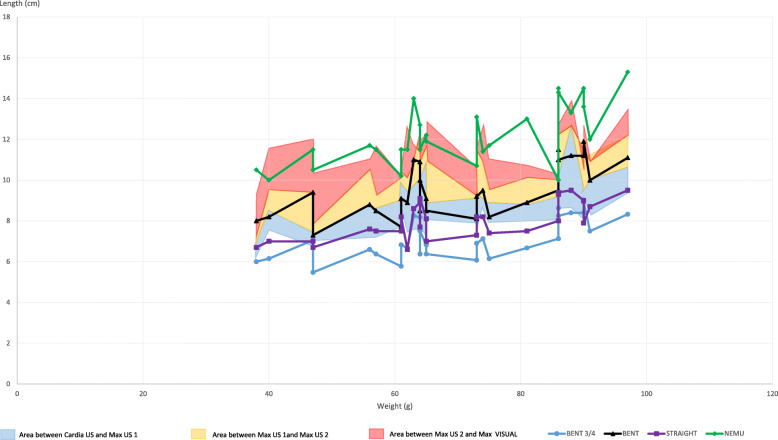
Tube lengths of BENT ¾, BENT, STRAIGHT and NEMU (in cm) with respect to weight (in g) in cats. Blue area: area between CARDIA US and Max US1. Orange area: area between Max US1 and Max US2. Red area: area between Max US2 and Max VISUAL. Values of BENT ¾ and STRAIGHT, found below the blue area (area defined by Cardia US and Max US1), are in the esophagus. Values found in the blue, orange, red areas or above are found in the stomach or further (looped, perforated or in the duodenum). BENT was never found below the blue area, therefore never in the oesophagus

CARDIA US, MAX US1, MAX US2 and MAX VISUAL of all neonates are shown in Fig. 4. In most BENT ¾ (84%) and in a few STRAIGHT (36%) measurements, the tip of the tube was located in the esophagus, as shown in Fig. 4, below the blue area. In a few BENT ¾ (16%), in half of BENT (50%) and in most STRAIGHT (61%) measurements, the tip of the tube was found in the stomach, i.e.*,* in the blue area. One case of STRAIGHT (3%), half of BENT (50%) and all NEMU cases were found further away than MAX US1, as shown in Fig. 4, in the orange and red areas or above.

To assess the reliability of ultrasonography versus visual analysis, the mean difference between CARDIA US and CARDIA VISUAL was determined as − 0.04 ± 0.31 cm (range − 0.6 to 0.7 cm) (*P* = 0.47). BENT exceeded MAX VISUAL in one kitten, while BENT ¾ and STRAIGHT did not exceed MAX VISUAL in any of the kittens.

After the measurement of CARDIA VISUAL and MAX VISUAL, we attempted to force the tube to go further than MAX VISUAL in 14 kittens, leading to looping of the tube in 8 kittens (Fig. 3c), perforation in 4 kittens (Fig. 3d), or the impossibility to force further than MAX VISUAL in 2 kittens.

### Tube measurements in puppies

**Fig. 5:**
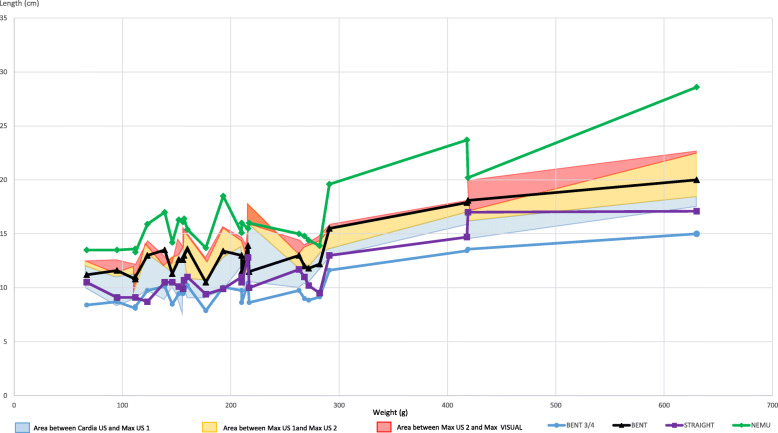
Tube lengths of BENT ¾, BENT, STRAIGHT and NEMU (in cm) with respect to weight (in g) in puppies. Blue area: area between CARDIA US and Max US1. Orange area: area between Max US1 and Max US2. Red area: area between Max US2 and Max VISUAL. Values of BENT ¾ and STRAIGHT, found below the blue area, are in the oesophagus. BENT was never found below the blue area, therefore never in the oesophagus

CARDIA US, MAX US1, MAX US2 and MAX VISUAL are shown together with BENT ¾, STRAIGHT, BENT and NEMU in Fig. 5. To evaluate the optimal tube length (= with the tip within the stomach while it is empty or full, without any risk of perforation), BENT, BENT3/4, STRAIGHT and NEMU were compared to CARDIA US to evaluated whether the tip was within the stomach. Then, these measurements were compared to MAX US1, evaluating whether the tip was within the empty stomach without risk of perforation and to MAX US2, evaluating whether the tip was within the full stomach without risk of perforation.

We next assessed whether BENT ¾ was in the stomach or the esophagus. We observed that BENT ¾ was smaller than CARDIA US and was in the esophagus in 18 cases. BENT ¾ was at a mean − 0.99 ± 1.27 cm (range − 2.75 to 1.55 cm) cranial to the cardia.

To assess whether STRAIGHT was positioned close to the cardia, the mean difference between STRAIGHT and CARDIA US was determined as 0.23 ± 1.28 cm (range − 2.4 to 3.3 cm) (*P* = 0.37). There were no differences between the groups of fresh and frozen puppies (*p* = 0.08). STRAIGHT was longer than CARDIA US in 15/25 puppies and in the esophagus in 10/25 puppies. STRAIGHT exceeded MAX US1 in 2 puppies, and no measurements exceeded MAX US 2 (Table 1).

BENT was longer than CARDIA US in all 25 puppies, with a mean 2.3 ± 1.3 cm (range 0.3 to 4.7 cm) difference. BENT exceeded MAX US1 and MAX US2 in five and four cases, respectively (Table 1).

NEMU was longer than most of the maximal measurements, with a mean of 5.56 ± 2.00 cm (range 2 to 11 cm) distance from the cardia.

To assess the reliability of ultrasonography versus visual analysis, the mean difference between CARDIA US and CARDIA VISUAL was examined, which was 0.20 ± 0.58 cm (range − 0.9 to 1.5 cm) (*P* = 0.10). The groups of fresh and frozen puppies were not significantly different (*p* = 0.62). BENT exceeded MAX VISUAL in one puppy, while BENT ¾ and STRAIGHT did not exceed MAX VISUAL in any of the puppies.

### Formula of cardia placement based on weight

**Fig. 6:**
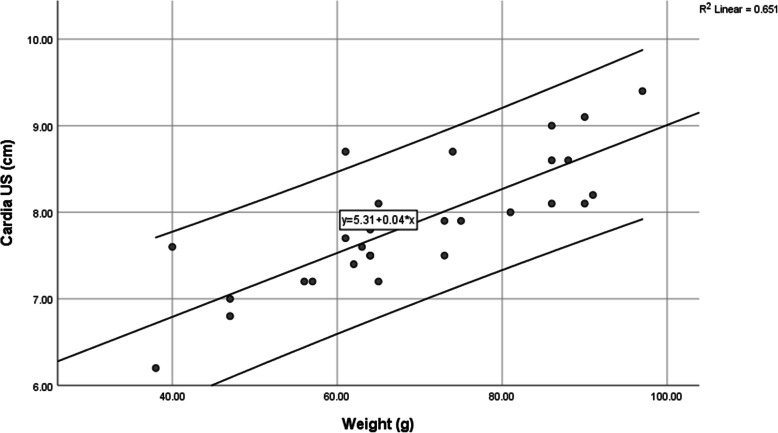
Linear correlation between weight and Cardia in kittens less than 1 day old, with upper and lower 95% confidence intervals

**Fig. 7:**
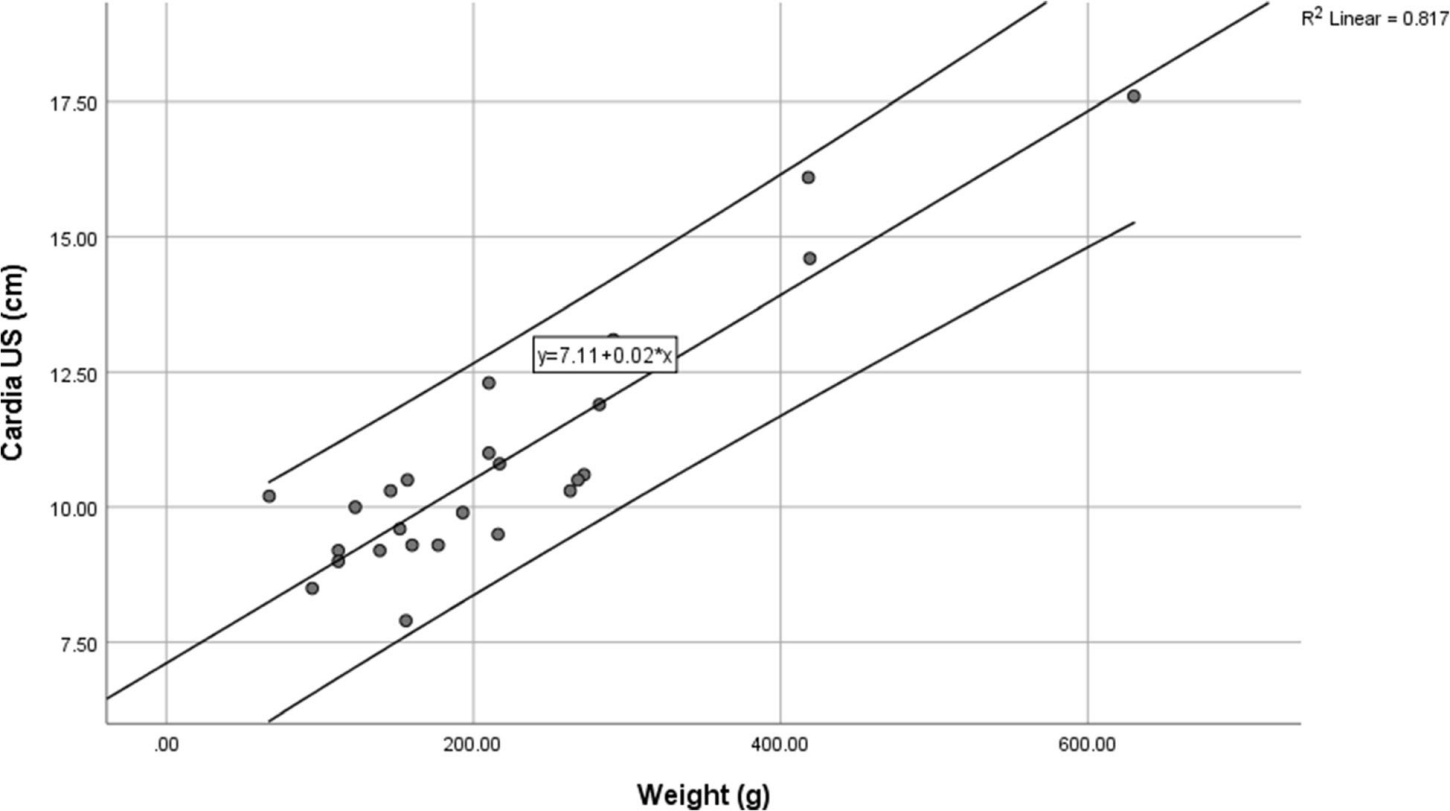
Linear correlation between weight and cardia in puppies less than 1 day old, with upper and lower 95% confidence intervals

The position of the cardia follows a linear regression compared to weight, with a strong correlation in cats (*r*^2^ = 65%) (Fig. 6) and puppies (*r*^2^ = 81%) (Fig. 7). The formulas, where Y_cardia_ is the length of the tube to reach the Cardia, and X the weight of the neonate, are as follows:

$$ {\mathrm{Y}}_{\mathrm{cardia}}=5.3+3.7\ast \mathrm{X}/100\ \mathrm{in}\ \mathrm{kittens} $$

$$ {\mathrm{Y}}_{\mathrm{cardia}}=7.1+1.7\ast \mathrm{X}/100\ \mathrm{in}\ \mathrm{puppies} $$

This equation predicts the length of insertion to reach the cardia. Using this formula on the 28 kittens and 25 puppies of the study, the tube was placed in the stomach in 43 and 48% of cases in kittens and puppies respectively (Table 1).

To avoid oesophageal placement 1.96 standard deviations of the means were added to the formula. The standard deviation for kittens and puppies for the weight was 0.005 and 0.002, respectively, and for the length of the cardia, it was 0.378 and 0.421, respectively. Adding 1.96 standard deviations to the previous formula allows to place the tube beyond the cardia (in the stomach) with 97.5% confidence (95% of the area of a normal distribution is within *1.96 standard deviations* of the mean).

$$ {\mathrm{Y}}_{\mathrm{stomach}}=6+4.7\ast \mathrm{X}/100\ \mathrm{in}\ \mathrm{kittens} $$

$$ {\mathrm{Y}}_{\mathrm{stomach}}=8+2.1\ast \mathrm{X}/100\ \mathrm{in}\ \mathrm{puppies} $$

Using the formula on the 28 kittens in this study, all the tubes were placed beyond the cardia, but 13 values exceeded MAX US1, three values exceeded MAX US2 and zero MAX VISUAL. In the 25 puppies, 11 values exceeded MAX US1, one MAX US2 and zero MAX VISUAL (Table 1).

### Stomach volume

The maximal volume of the stomach in puppies was found to be 1.56 ± 1.28 mL (range 0.30 to 3.66 mL) by ultrasonography and 2.04 ± 1.28 mL (range 0.51 to 5.59 mL) when measured visually. In mL per 100 g of body weight, the maximal volume of the stomach was 1.10 ± 0.87 mL/100 g of body weight (range 0.09 to 2.88 mL/100 g) by ultrasonography and 1.20 ± 0.57 mL/100 g of body weight (range 0.33 to 2.61 mL/100 g) when measured visually.

The maximal volume of the stomach in kittens was 1.10 ± 0.60 mL (range 0.14 to 2.34 mL) by ultrasonography and 1.34 ± 1.00 mL (range 0.46 to 4.81 mL) when measured visually. In mL per 100 g of body weight, the maximal volume of the stomach was 1.55 ± 0.86 mL/100 g of body weight (range 0.30 to 3.65 mL/100 g) by ultrasonography and 1.95 ± 1.21 mL/100 g of body weight (range 0.51 to 5.60 mL/100 g) when measured visually.

## Discussion

Studies on live canine and feline neonates concerning proper tube feeding placement are lacking, likely due to the dearth of ethical methods to analyze tube placement and its complications. To provide new insights into tube feeding management in canine and feline neonates, we used deceased kittens and puppies, accepting that the absence of muscular tone and fragility of the stomach wall may somewhat bias the results. This study demonstrated that in a clinical setting, appropriate placement of the tube can be performed quickly using ultrasonography: stomach wall deformation and stomach volume were accurately visualized, even without clipping hairs. Although no difference was found between frozen and fresh puppies, further validation is required in live animals to confirm these results.

Despite these promising findings, ultrasonography cannot be performed in a home setting. Therefore, we additionally compared different anatomical predictive markers previously described in human infants and/or in puppies and kittens to determine the optimal length for tube feeding with respect to the location of the tip of the tube in canine and feline neonates up to 1 day of age. The description of tube placement using BENT ¾, STRAIGHT, BENT or NEMU aimed to assess potential risks to the integrity of the gastric wall and to determine whether the tube was placed in the esophagus, in the stomach or further. However, the study on dead puppies cannot provide an answer to whether stomach feeding or esophageal feeding is preferable. Both techniques are used in adults [28] without significant differences in complication rates [29], however in most cases CRI feeding was performed. Bolus feeding in neonates with the tube end placed in the distal oesophagus might increase the risk of reflux, on the other hand in adults, forcing a feeding tube into the stomach might induce sphincter incompetence due to feeding tubes passing through the lower oesophageal sphincter [47]. The tips of feeding tubes are mostly presented with lateral openings. Using a tube with the flared end trimmed may reduce regurgitation risks, as previously suggested [6]. To avoid mucosal injuries due to sharp edges, a flame may be used to slightly melt the plastic, smoothing the edges. Flamed-ended feeding tubes might still be traumatic and more difficult to use. Therefore, we suggest using readily open-ended soft tubes aiming to reduce the risk of residual milk left in the oesophagus after extubation. A drop of milk intended to be used for the feeding procedure or water soluble veterinary lubricant may be used to grease the tube before insertion [7]. Reduced contractility of the gastrointestinal tract was observed in human preterm neonates [48, 49] and in canine neonates, with a progressive increase in contractility occurring 3–7 days after birth [50]. Canine gastrointestinal maturation is an evolutionary process, and gastrointestinal barrier closure is completed very early, 16–24 h after birth [51]. Later, during the nursing period, the stomach increases approximately twofold in relative weight [52], implying that significant changes in the gastrointestinal tract occur within the first days of life. It is therefore very important that our results, obtained by studying dead neonates up to the age of 24 h, should not be extrapolated in any way to older puppies and kittens. Additional studies investigating changes in stomach size, as well as gastric emptying in growing neonates, might improve tube feeding strategies.

BENT was the only measurement that avoided esophageal intubation in 100% of kittens and puppies; therefore, this length may be used for gastric emptying [2]. However, in most cases, gastric emptying is performed easily and quickly by lowering the neonate’s head and aspirating the oropharyngeal and/or esophageal areas with a nose pump, as it is performed to clear the airways immediately after delivery [53]. Stomach emptying might be beneficial to reduce diaphragmatic pressure during neonatal resuscitation [2], as well as to remove clotted milk in hypothermic neonates (author’s personal experience). However, concerning injury risks, tube placement with BENT was not always harmless because BENT exceeded MAX US1, MAX US2, and MAX VISUAL in 19, 8 and 2 neonates, respectively (Table 1). We define tube misplacement here as measurements exceeding MAX US1. Using rigid tubes, such as 8 Fr adult feeding tubes, may induce stomach damage in neonates (Fig. 3d). It should be self-evident that forcing the tube to go further than the MAX VISUAL breakpoint should be avoided. This action induced stomach perforation in four kittens and looping in eight kittens (Fig. 3c), as described as previously reported complications [1, 5]. The authors do not recommend using 8Fr tubes in polyvinylchloride, such as the rigid ones commonly placed in adult dogs and cats, with BENT on neonates, although the incidence of gastric perforations in this study might be overestimated due to autolysis: the prevalence of perforation is considered to be 1.1% in low birth weight human neonates [20]. Using softer tubes [1, 6] may reduce the risk for complications, such as stomach injuries, although looping, regurgitation or kinking of the tube remain as possible additional risk factors [1, 5]. For the feeding of neonates, STRAIGHT seems more suitable, which was primarily found close to the area of the cardia, with 32/53 cases found in the stomach (60%). BENT ¾ might be used, even if it is found most often in the esophagus (45/53 cases; 85%). Concerning risks for injuries, STRAIGHT exceeded MAX US1 in three cases, while BENT ¾ never exceeded MAX US1. The authors conclude that gastric injuries are minimized by using either BENT ¾ or STRAIGHT, although the prevalence of regurgitations should be assessed in live animals. Measurements with NEMU are not appropriate in puppies or kittens due to an excessive intubation length (Table 1), in contrast to preterm infants [54]. Concerning the diameter used for tube feeding, some recommend a 5 Fr diameter for < 300 g neonates and 8 Fr for > 300 g animals [21–23]. Others discourage the use of small catheters due to the increased risks for looping [1], and some recommend using the largest tube that easily passes [4] to decrease the chance of inadvertent endotracheal intubation. Further studies are needed on this subject to reach a final consensus. Vocalization is a good indicator for appropriate intubation in the esophagus versus in the trachea, although some puppies might be too weak to cry during the intubation process. Minimal restraint is also suggested to decrease the risks for aspiration or other feeding accidents [1], while body positioning may also promote gastric emptying and reduce gastroesophageal reflux, as shown in human preterm infants [55]. Elevating the carnivores’ forequarters or holding the head elevated during feeding may simulate natural nursing and help to maintain tube placement [1]. In our study, the intubation procedure and ultrasonography were performed in dorsal recumbence. To be able to perform ultrasonography on live moving neonates, we suggest inserting the tube as previously described in the most physiological position possible with minimal restraint [1], maintaining the tube with the head with one hand and elevating the forequarters to make way to the abdomen for the ultrasound operator. On four puppies and on three kittens, ultrasound was performed in dorsal, ventral recumbence and with the forequarters elevated at 45°, the variations on CARDIA US and MAX US 2 were minimal (data available on request). The identification of the cardia using these three positions is feasible.

During measurements performed in this study, it became clear that without ultrasound, a residual risk always remains with tube insertion in up-to-one-day-old puppies and kittens. Since the position of the cardia can be predicted very well using body weight, as shown by the strong correlation rate in cats (*r*^2^ = 65%) (Fig. 6) and dogs (*r*^2^ = 81%) (Fig. 7), we recommend the use of weight-based formulas to determine tube length. Whenever this formula is used in puppies and kittens, values exceeding maximum measurements are reduced compared to BENT (Table 1), indicating that this formula may avoid extreme measurements, which are precisely those that may induce trauma.

The maximal stomach volume was found to be 1.2 and 1.9 mL per 100 g of body weight in feline and canine neonates, respectively. This contradicts most previous feeding instructions with a maximum amount of 4–5 mL milk replacer/100 g of bodyweight [5, 6, 11, 21, 22] but is in agreement with data found on 4 newborns in an original study by Andersen [52]. Even though the maximum stomach volume and the maximum feeding quantity are not identical, they are closely related. Factors that further influence feeding quantity are, among others, the duration and frequency of feeding, as well as the gastrointestinal clearance and motility in relation to the temperature of the neonate [2, 56]. Safety rules should be adapted according to age to avoid overfeeding and regurgitation. Nevertheless, our model has several limitations. Tonicity of the pyloric sphincter, esophagus, and stomach compliance differ between live and dead animals; therefore, these results should be replicated in live animals with the necessary precautions.

The great benefit of our study is that different feline and canine breeds of various sizes, including normo-, brachy- and dolichocephalic breeds, were represented in our study population, while previous data were generated only in beagles [52]. Nevertheless, despite this large morphological variance, a clear linearity between weight and cardiac distance was observed. Similar results have been obtained in adult dogs [57]. This indicates that our results might be applied to less-than-one-day-old neonates of a wide variety of breeds, although the use of cadavers remains a clear limitation.

BENT and STRAIGHT measurements were performed on dead neonates, while BENT ¾ was calculated based on the values of BENT. The variations found in this study are therefore underestimated compared to live, moving animals, increasing the potential discussed risks for injuries in vivo. Using the equation presented herein reduces the risk to neonates because the probability of the tube being in the stomach is increased.

## Conclusions

Ultrasonography is a reliable tool for correct gastric tube placement in canine and feline neonates. However, whenever ultrasonography is not available, the proposed weight-based formula is a good option for choosing the correct length of tube insertion. In addition to the possible complications of tube feeding, it is important to be aware of the reduced stomach capacity in less-than-one-day-old neonates. Additional studies are required to assess regurgitation risks with respect to esophageal versus gastric intubation in canine and feline neonates.

## Data Availability

The datasets generated and/or analyzed during the current study are available from the corresponding author on reasonable request.

## Abbreviations

BENT: Length of the tube, held along the outside puppy’s body, bent, from behind the last rib, to the tip of the nose
BENT ¾: Three-quarters of the length of the tube, held along the outside puppy’s body, bent, from behind the last rib to the tip of the nose
STRAIGHT: Length of the tube, held along the outside puppy’s body, straight, from behind the last rib, to the tip of the nose
NEMU: Nose-Earlobe/Pinna-Mid Umbilicus measurement
CARDIA US: Length of the tube until it reaches the cardia by ultrasonography
CARDIA VISUAL: Length of the tube until it reaches the cardia, visually after coeliotomy
MAX US 1: Length of the tube until it deforms the wall of an empty stomach by ultrasonography
MAX US 2: Length of the tube until it deforms the wall of a full stomach by ultrasonography
MAX VISUAL: Length of the tube until it deforms the wall of a full stomach, visually, after coeliotomy
